# Transmission of *Mycobacterium tuberculosis* in schools: a molecular epidemiological study using whole-genome sequencing in Guangzhou, China

**DOI:** 10.3389/fpubh.2023.1156930

**Published:** 2023-05-11

**Authors:** Ying Lin, Yuhua Du, Hongcheng Shen, Yangfeng Guo, Ting Wang, Keng Lai, Danni Zhang, Guangmin Zheng, Guifeng Wu, Yu Lei, Jianxiong Liu

**Affiliations:** ^1^Department of Tuberculosis Control, Guangzhou Chest Hospital, Guangzhou, China; ^2^Department of Preventive Health Care, Guangzhou Chest Hospital, Guangzhou, China; ^3^Guangzhou Primary and Secondary School Health and Health Promotion Center, Guangzhou, China; ^4^Academy of Public Health, Guangzhou Medical University, Guangzhou, China; ^5^State Key Laboratory of Respiratory Disease, Guangzhou, China

**Keywords:** whole-genome sequencing, epidemiological study, cluster analysis, phylogenetic analysis, multidrug-resistant *Mycobacterium tuberculosis*, school

## Abstract

**Background:**

China is a country with a high burden of tuberculosis (TB). TB outbreaks are frequent in schools. Thus, understanding the transmission patterns is crucial for controlling TB.

**Method:**

In this genomic epidemiological study, the conventional epidemiological survey data combined with whole-genome sequencing was used to assess the genotypic distribution and transmission characteristics of *Mycobacterium tuberculosis* strains isolated from patients with TB attending schools during 2015 to 2019 in Guangzhou, China.

**Result:**

The TB incidence was mainly concentrated in regular secondary schools and technical and vocational schools. The incidence of drug resistance among the students was 16.30% (22/135). The phylogenetic tree showed that 79.26% (107/135) and 20.74% (28/135) of the strains belonged to lineage 2 (Beijing genotype) and lineage 4 (Euro-American genotype), respectively. Among the 135 isolates, five clusters with genomic distance within 12 single nucleotide polymorphisms were identified; these clusters included 10 strains, accounting for an overall clustering rate of 7.4% (10/135), which showed a much lower transmission index. The distance between the home or school address and the interval time of symptom onset or diagnosis indicated that campus dissemination and community dissemination may be existed both, and community dissemination is the main.

**Conclusion and recommendation:**

TB cases in Guangzhou schools were mainly disseminated and predominantly originated from community transmission. Accordingly, surveillance needs to be strengthened to stop the spread of TB in schools.

## 1. Introduction

Tuberculosis (TB), caused by *Mycobacterium tuberculosis*, is a global public health concern. In 2021, 10.6 million people worldwide were affected with TB; of these, 7.36% cases were from China, with an estimated incidence rate of 55.2 per 100,000 individuals, leading to China having the third highest TB case burden globally (1). A large number of people moving through the community increases the susceptibility to disease outbreaks. In China, TB outbreaks are common in educational settings such as high schools and universities (2). Data from the Chinese Center for Disease Control and Prevention showed that more than 45,000 students were diagnosed with pulmonary TB nationwide in 2020, with an incidence rate of 15.9 per 100,000 individuals (3). Schools are places with highly concentrated crowds. Once TB occurs, it is easily transmitted and may eventually cause an epidemic. TB epidemics in schools not only harm the health of students but also affect the teaching–learning process.

TB has been frequently reported among students in recent years (4, 5). However, relevant studies have mainly focused on traditional epidemiological characterization, risk factor analysis for morbidity, and outbreak management evaluation. We feel it is important to ascertain the key factors for TB prevention, control, and transmission in schools; finding out the source of infection and clarifying the mode of transmission may be beneficial. Molecular epidemiological studies have unique advantages in transmission chain investigation and strain tracing in TB. Whole-genome sequencing (WGS) is currently the most accurate method for molecular epidemiological studies on TB (6). Compared with IS6110 restriction fragment length polymorphism typing and mycobacterial interspersed repetitive unit–variable number of tandem repeat typing, WGS provides an improved resolution of microbial correlation among isolates. Moreover, the microbial correlation between cases can be further refined by using WGS, which helps clarifying whether the school TB cases were a result of previous transmission or recent transmission. Chains of transmission can also be identified using WGS combined with epidemiological data.

Guangzhou is a city in southern China with a population of 18.74 million; of this population, 3.1 million (16.57%) individuals are school students (data from the local education department excluding technical school students), and accounting for the major proportion of students in China (235/1,412, 16.64%) according to the National Bureau of Statistics of China (2021) (7, 8). Moreover, the reported rate of pulmonary TB ranged between 15.74/100,000 and 20.63/100,000 among students in Guangzhou from 2014 to 2018, which is close to the overall rate in China (13.39/100,000 to 17.97/100,000) (9, 10). In addition, the Guangzhou Chest Hospital established a TB strain bank in 2013; this bank hosts a comprehensive collection of *Mycobacterium tuberculosis* strains isolated from patients with TB diagnosed in Guangzhou. The Guangzhou Chest Hospital also functions as a TB management institution in Guangzhou and is responsible for the management of TB epidemics in schools; it has data on epidemiological surveys of school students with TB. Guangzhou city is therefore an ideal site for studying the transmission of *Mycobacterium tuberculosis* in schools.

In the present study, we used WGS combined with epidemiological surveys to ascertain the genotypic distribution and transmission characteristics of TB strains isolated from schools between 2015 and 2019 and to clarify the transmission patterns of TB in schools. Our study findings may unveil the key factors for TB prevention and control in schools and help formulate effective strategies for preventing and controlling TB outbreaks in schools in Guangzhou city and throughout China.

## 2. Methods

### 2.1. Sample source

Active *Mycobacterium tuberculosis* cases registered in Guangzhou city from 2015 to 2019 with the occupation of teachers, students, and children in childcare were enrolled in our study. We divided them into two categories: positive and negative sputum cultures patients.

As Guangzhou Chest Hospital was the assigned and largest tuberculosis hospital in Guangzhou city, TB was diagnosed and tread at it principally. Patients submitted their sputum samples for medical testing during their treatment at the Guangzhou Chest Hospital; the hospital deposited their strains in the strain bank for scientific research after obtaining informed consent from the patients. In our study, the strains fit the selection criteria above were identified from the strain bank and re-cultured to isolate active strains for whole-genome sequencing.

The patients' basic and clinical treatment data were extracted from the Chinese Center for Disease Control and Prevention and the hospital information system of the Guangzhou Chest Hospital. The study protocol was approved by the Ethics Review Committee of the Guangzhou Chest Hospital (No. 2019045).

### 2.2. Epidemiological analysis

We divided the patients whom enrolled in our study into different groups: active TB group, sputum culture-positive TB group, and whole genome sequencing group, and performed statistical analysis based on information of gender, age, occupation school, type, patient source, and TB history.

Due to the limitations in strain preservation and re-culture, we cannot performed WGS with all sputum culture-positive strains. To control the bias caused by small sequencing samples, this study additionally performed clustering analysis of all sputum culture-positive patients by traditional epidemiological methods to understand the prevalence of TB. According to the norms of school tuberculosis control in China, school TB epidemic was calculated based on school semester [one semester (including holidays) is 6 months]. Due to the huge size of university in Guangzhou, students from different collages intersected less. Therefore, in this study, colleges were used as the unit of measurement for university TB epidemics, while primary school, regular secondary schools and vocational-technical colleges TB epidemics were calculated with the whole school students. If diagnosis dates between two patients in the same school (college) was <6 months, the two patients were considered to have a possible epidemiological association.

### 2.3. WGS

#### 2.3.1. Genomic DNA extraction and sequencing

We subjected 400 μl of inactivated *Mycobacterium tuberculosis* culture to ultrasonic dispersion. DNA was extracted using a modified cetyltrimethylammonium bromide (CTAB) method, and the extracted DNA was then subjected to purity and concentration measurements using an ultra-micro spectrophotometer (Thermo NanoDrop 2000) and a Qubit 3.0 Fluorimeter, respectively. After quantification and quality testing, the extracted genomic DNA was broken down into 200–300 bp nucleic acid fragments using an ultrasonic interrupter (Covaris). These fragments were then used to build DNA libraries, which were subjected to concentration and length distribution measurements using a Qubit 3.0 Fluorimeter and the Agilent 2100 Bioanalyzer, respectively. Only those DNA libraries that passed the measurement were pooled according to their effective concentration and target data volume. The selected DNA libraries were then operated using a paired-end sequencing program (PE150) on the Illumina sequencing platform.

#### 2.3.2. Sequencing data analysis

The sequencing data of *Mycobacterium tuberculosis* strains were analyzed using the sequence analysis of *Mycobacterium Tuberculosis* platform (https://samtb.uni-medica.com), as previously described (11). In the first step, the Sickle tool was used to trim the WGS data, and sequencing reads with a Phred base quality >20 and read length >30 were used for further analysis (12). The retained sequencing reads were mapped to the reference genome using Bowtie2 (v2.4.1), and the SAMtools (v1.6)/VarScan (v2.3.6) suite was used for calling single nucleotide polymorphisms (SNPs) with a mapping quality >30 (13). In the second step, the processed high-quality sequence data were compared with the H37Rv *Mycobacterium tuberculosis* reference genome, and samples with a sequencing depth >20× and genome coverage >95% were selected based on the comparison results. In the third step, single-sample variant assays were performed separately for each strain to understand the genotypes and mutations in drug resistance genes. Referring to similar studies (14, 15), the strains were classified into different lineages (L) and sublineages; L2 strains can generally be divided into L2.1, L2.2, and L2.3 sublineages, with L2.1 and L2.2 representing the ancient Beijing type and L2.3 representing the modern Beijing type (16). Resistance-associated mutations were found, which indicated that the study samples were resistant to 17 antituberculosis drugs reported previously (17): isoniazid, rifampicin, ethambutol, pyrazinamide, streptomycin, amikacin, capreomycin, kanamycin, moxifloxacin, ofloxacin, ethionamide, para-aminosalicylic acid, cycloserine, linezolid, clofazimine, bedaquiline, and delamanid.

Mono-resistance was defined as resistance to only one anti-tuberculosis drug. Multidrug-resistant (MDR) TB was defined as TB caused by isolates with mutations conferring resistance to at least isoniazid and rifampicin. Pre-extensively drug-resistant (pre-XDR) TB was defined as TB caused by an MDR strain with mutations conferring additional resistance to fluoroquinolones or any second-line injected drugs, and extensively drug-resistant (XDR) TB was defined as TB caused by an MDR strain with mutations conferring resistance to both of these antibiotic classes (13).

In the fourth step, phylogenetic trees (18) were constructed using the maximum likelihood method. Bootstrap analysis was run 500 times and visualized using Interactive Tree of Life software (https://itol.embl.de/). Based on Holt KE's principles (19), the terminal branch lengths (TBLs) of the different strains were calculated, and multiple strains with SNP distances ≤ 12 and on the same branch of the evolutionary tree were defined as clustered strains (14, 15). Moreover, the epidemiological associations among the clustered strains were analyzed in conjunction with the epidemiological findings.

### 2.4. Statistical analyses

Continuous variables were represented as the mean ± standard deviation, whereas categorical variables were represented as medians and interquartile ranges. Group differences were compared using the chi-square test and Non-parametric tests (*K* independent sample), and *P* < 0.05 was considered statistically significant. All statistical analyses were conducted using SPSS v25.0 (IBM, USA). Missing data were not included in the analysis. TBL distribution maps of the sublineage strains and geographic markers of strains were drawn using Python 3.9.

### 2.5. Role of the funding source

The sponsors were not involved in the study design, data collection, data analysis, data interpretation, or manuscript drafting. The corresponding author had full access to all the study data as well as final responsibility in terms of the decision to submit the manuscript for publication.

## 3. Results

### 3.1. General information

**Figure 1:**
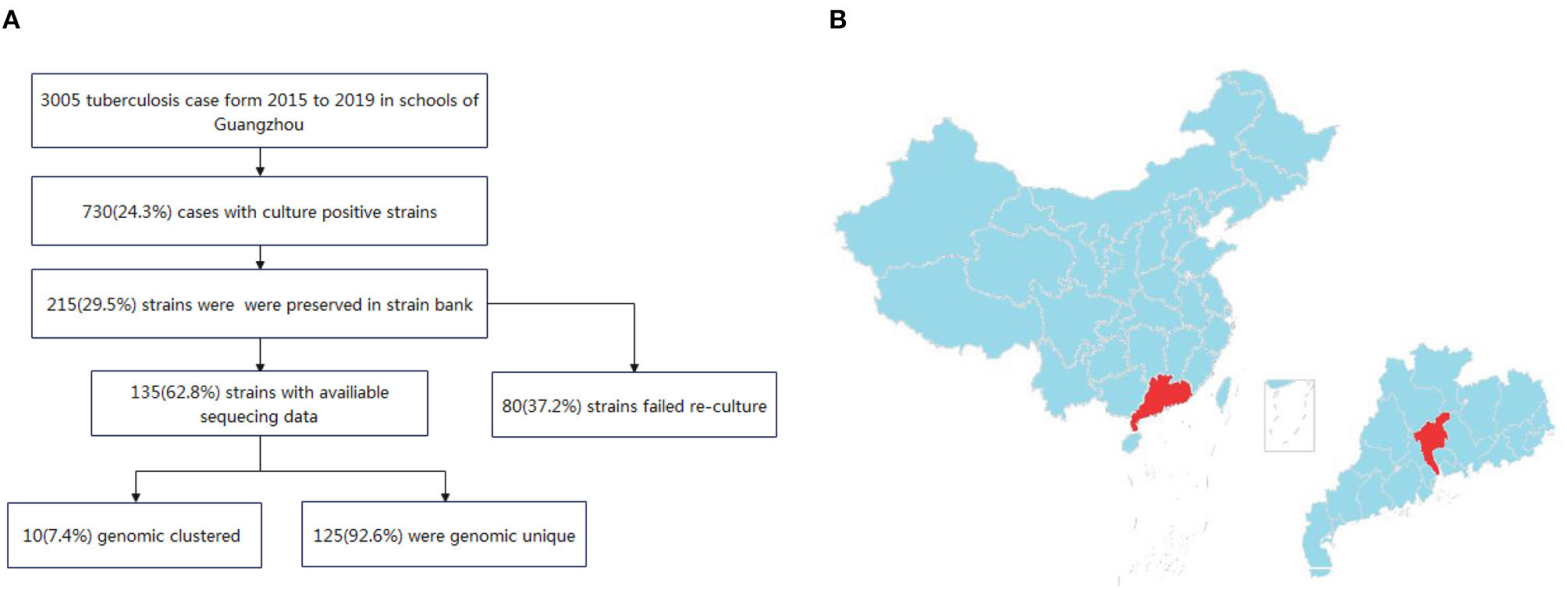
**(A)** Flow chart of the isolates included in the study. **(B)** Map of the study site in Guangzhou, China. The study site is located in central Guangdong province, which is part of the southern coastal region of China.

Between January 2015 and December 2019, a total of 3,005 patients in schools with active pulmonary TB were registered in Guangzhou city. Of these patients, 730 had positive sputum cultures. 29.5% (215/730) were preserved in the strain bank of Guangzhou Chest Hospital. Finally, 62.8% (135/215) of the sputum specimens were re-cultured from frozen stocks and completed the whole-genome sequencing, while 80 strain were failed to re-cultured (Figure 1).

The school-based patients with TB registered in Guangzhou mainly belonged to the 16–18 and 19–24 year age groups. Compared with patients with negative sputum culture, those with positive sputum culture comprised a larger proportion in the age group of <15 years, and the difference was statistically different (χ^2^ = 41.99, *P* < 0.001). There were 18.5% (135/730) of patients with positive sputum culture patients had whole-genome sequences. The age composition of patients with and without whole-genome sequences was not statistically significantly different (χ^2^ = 7.74, *P* = 0.102). Moreover, there were no statistically significant differences in the gender and occupation compositions of the patients with TB between the positive and negative sputum culture groups and between patients with and without whole-genome sequences [χ_gender(sputumculture)_^2^ = 0.6, *P* = 0.437; χ_gender(WGS)_^2^ = 0.98, *P* = 0.320; χ_occupation(sputumculture)_^2^ = 1.51, *P* = 0.219; χ_occupation(WGS)_^2^ = 2.06, *P* = 0.152] (Table 1).

**Table 1:** Demographic and clinical characteristics of the school-based patients with TB in Guangzhou from 2015 to 2019.

	**All registered patients (*N* = 3,005) No. (%)**	**Sputum culture-positive patients (*N* = 730) No. (%)**	χ^2*^	*P*-value^*^	**Patients with whole-genome sequences (*N* = 135) No. (%)**	**χ^2#^**	***P*-value^#^**
**Demographic characteristics**							
**Gender**							
Male	1,688 (56.17%)	401 (54.93%)	0.60	0.437	69 (51.11%)	0.98	0.320
female	1,317 (43.83%)	329 (45.07%)			66 (48.89%)		
**Age (years)**							
≤ 12	105 (3.49%)	33 (4.52%)	41.99	< 0.001	6 (4.44%)	7.74	0.102
13–15	238 (7.92%)	84 (11.52%)			21 (15.56%)		
16–18	900 (29.95%)	236 (32.33%)			52 (38.52%)		
19–24	1,447 (48.15%)	355 (45.89%)			49 (36.30%)		
≥25	315 (10.48%)	42 (5.75%)			7 (5.19%)		
**Occupation**							
Teacher	261 (8.69%)	47 (6.44%)	1.51	0.219	5 (3.70%)	2.06	0.152
Student	2,744 (91.31%)	583 (79.86%)			130 (96.30%)		
**School type**							
Child care facilities	74 (2.46%)	23 (3.15%)	50.03	< 0.001	0 (0%)	6.12	0.106
Primary	103 (3.43%)	39 (5.34%)			7 (5.19%)		
Regular secondary	837 (27.65%)	257 (35.21%)			54 (40.0%)		
Universities and colleges	1,196 (36.47%)	209 (28.63%)			31 (22.96%)		
Technical and vocational secondary	801 (29.98%)	202 (27.67%)			43 (31.85%)		
**Clinical factors**							
**Patient source**							
Health screening	276 (10.68%)	50 (6.85%)	130.84	< 0.001	7 (5.19%)	6.70	0.082
Doctor visits for illness	1,587 (53.81%)	526 (72.05%)			109 (80.74%)		
Referral	939 (28.99%)	119 (16.30%)			16 (11.85%)		
Follow-up	196 (6.52%)	35 (4.79%)			3 (2.22%)		
**Disease history**							
New patients	2,963 (98.60%)	718 (98.36%)	0.42	0.515	133 (98.52%)	0.03	0.869
Recurrence	42 (1.40%)	12 (1.64%)			2 (1.48%)		

* Statistical analysis (chi-square test) of the distributions between patients with positive and negative sputum cultures.

# Statistical analysis (chi-square test) of the distributions between patients with and without sequencing strains.

Regarding the school type, the overall proportion of regular secondary school students was much lower than that of the positive sputum culture patients (27.65 vs. 35.21%); by contrast, the proportion of university or college students was much higher than that of the positive sputum culture patients (36.47 vs 28.63%). This was attributable to the statistically significant differences between the patients with positive and negative sputum cultures (χ^2^ = 50.03, *P* < 0.001). However, there was no statistically significant difference between the positive sputum culture patients with and without whole-genome sequences (χ^2^ = 6.12, *P* = 0.106) (Table 1).

Furthermore, 10.68% of the patients were identified by health screening, including school entrance physical examinations or employee physical examinations; however, only 6.85% in the patients had positive sputum culture and 5.19% in the patients had whole-genome sequences. An increased number of patients with positive sputum cultures were identified by hospitalization, indicating that patients did not see a doctor until they feel sick. The source composition of sputum culture positive and negative patients was also statistically different (χ^2^ = 130.84, *P* < 0.001), but no statistically significant difference was noted between the positive sputum cultures patients with or without whole-genome sequences (χ^2^ = 6.70, *P* = 0.082). The majority of the patients with TB in schools were new cases, accounting for 98.60% (2,963/3,005) of all registered patients, 98.36% (718/730) of all sputum culture-positive patients, and 98.52% (133/135) of all patients with whole-genome sequences in our study. There were no statistically significant difference in any of the above subgroups (χ_sputumculture_^2^ = 0.42, *P* = 0.515; χ_WGS_^2^ = 0.03, *P* = 0.869) (Table 1).

### 3.2. Genotype and drug resistance analysis

**Figure 2:**
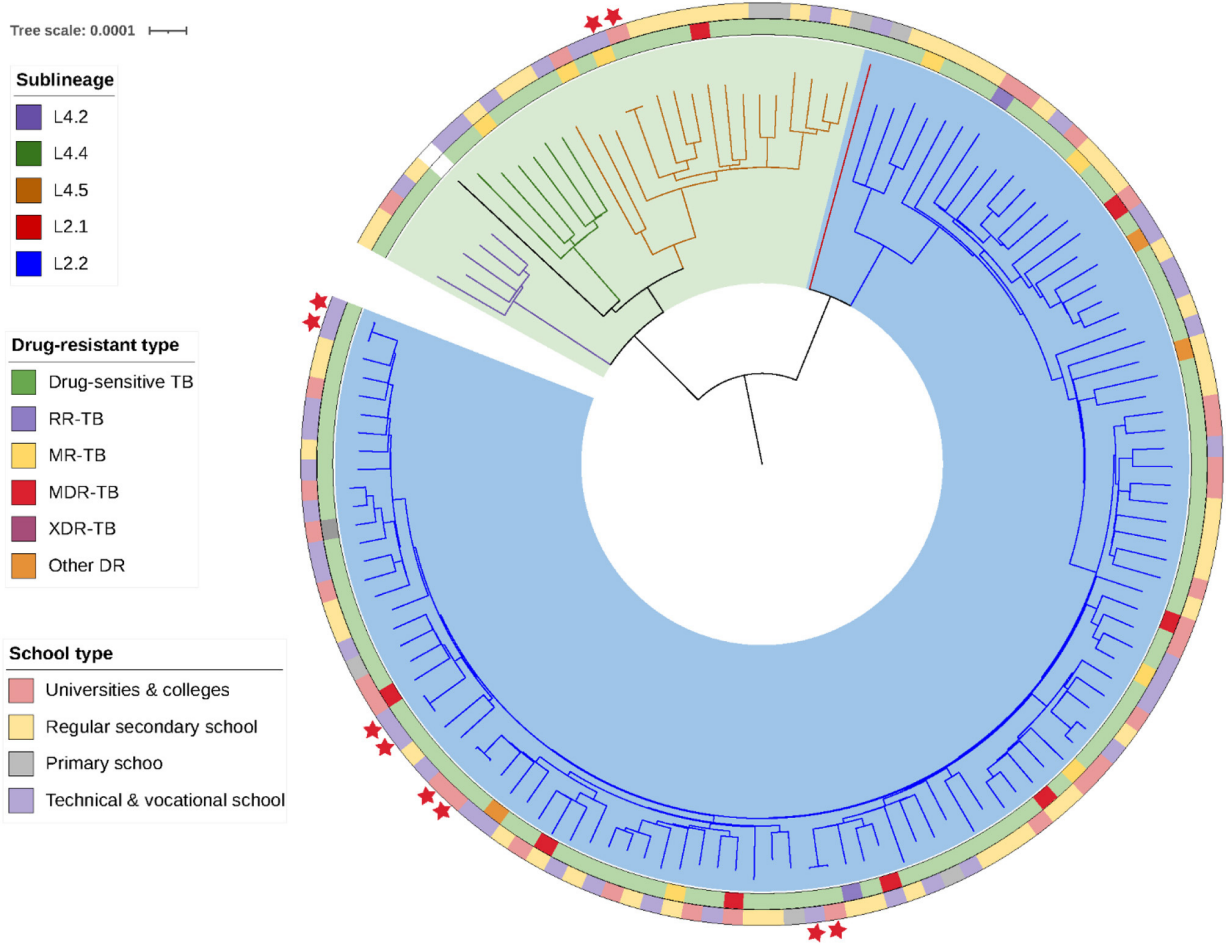
Phylogenetic tree of the 135 *Mycobacterium tuberculosis* strains isolated. The different colors on the branches indicate the different lineages and sublineages. The H37Rv *Mycobacterium tuberculosis* reference genome is represented in black.

**Figure 3:**
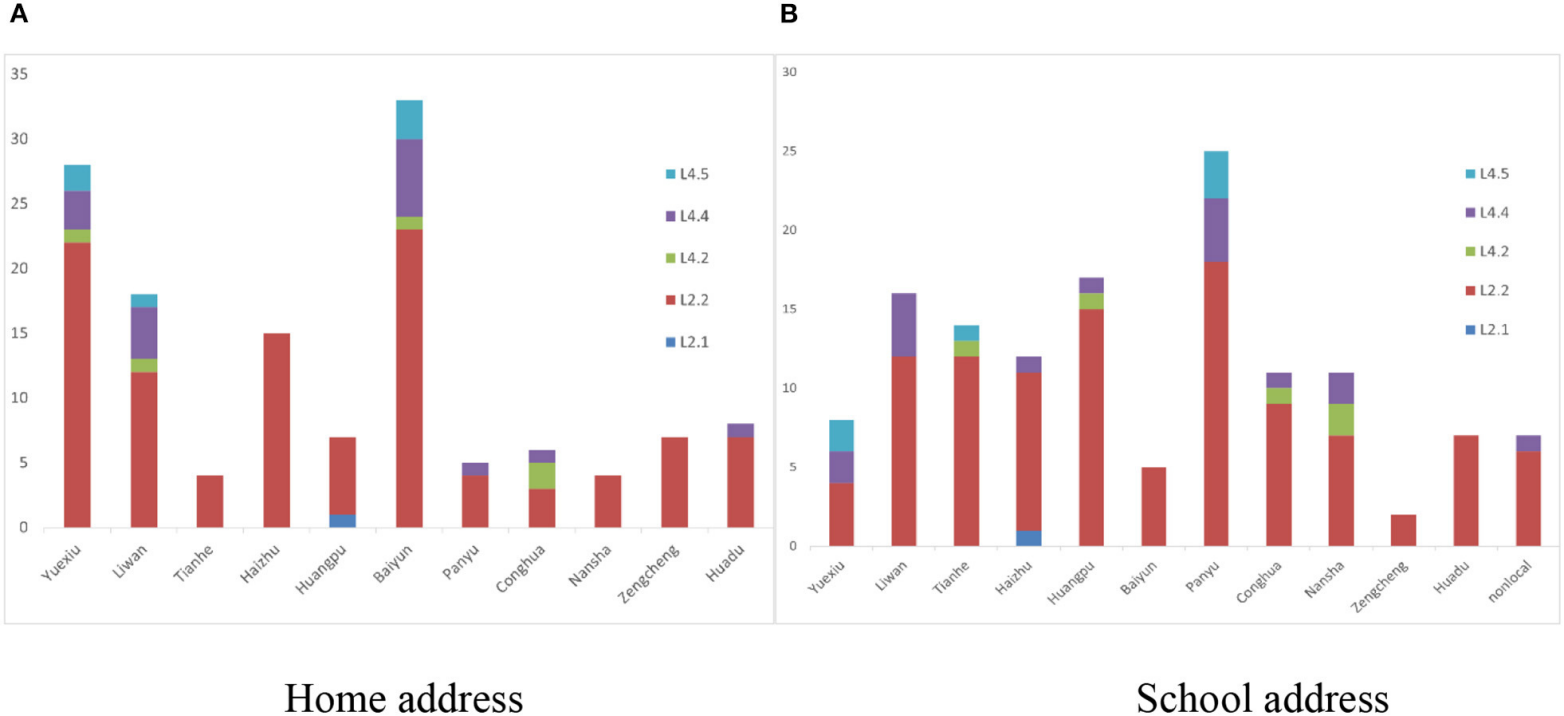
The distribution of the genotypes of 135 *Mycobacterium tuberculosis* in Guangzhou. **(A)** The distribution of the genotypes of school-based patients in different administrative regions by home address. **(B)** The distribution of the genotypes of school-based patients in different administrative regions by school address.

The whole genomes of the 135 *Mycobacterium tuberculosis* strains were sequenced with an average depth of 133X (114–164X) and average coverage of 99.34% (98.64–99.95%). Phylogenetic analysis showed that 79.3% (107/135) of the isolates predominantly belonged to the ancient Beijing family [including 99.1% (106/107) L2.2 and 0.9% (1/107) L2.1 strains]. All of the 20.7% (28/135) non-Beijing strains belonged to L4 [including 60.7% (17/28) L4.4, 21.4% (6/28) L4.5, and 17.9% (5/28) L4.2 strains] (Figure 2; Supplementary Table 1). The genotype distribution of school-based patients living in the Yuexiu, Liwan, and Baiyun districts was the same (χ^2^ = 4.006, *P* = 0.876); the genotype distribution of patients studying or working in the Yuexiu and Panyu districts was also the same (χ^2^ = 5.584, *P* = 0.061). However, the rest of the genotypes' distributions were significantly different (Figures 3A, B).

Analysis of the genome sequences of the mutations conferring resistance to 17 anti-tuberculosis drugs revealed that there were 113 (83.7%) pan-sensitive strains and 22 (16.3%) strains resistant to at least one anti-tuberculosis drug. Mono-resistant, MDR, and XDR strains accounted for 5.9% (8/135), 5.9% (8/135), and 0.7% (1/135) of all strains, respectively; the 8 MDR strains included five pre-XDR strains. Among the 17 anti-tuberculosis drugs, isoniazid has the highest rate of 11.11% (15/135), followed by streptomycin 8.15% (11/135), rifampicin 7.41% (10/135), and moxifloxacin and ofloxacin both 6.67% (9/135), respectively. No mutations associated with resistance to para-aminosalicylic acid, cycloserine, linezolid, clofazimine, bedaquiline, or dramani were detected (Figure 2; Supplementary Table 1).

Among the patients with positive cultures for these strains, the overall incidence of drug resistance was 16.30% (22/135), including 25.81% (8/31) among university students, which was higher than that noted among secondary school (16.36%, 9/55) and technical and vocational school (11.90%, 5/42) students; moreover, no drug resistance genes were detected in the TB strains isolated from the primary school students in this study. Except for that among primary school students, no statistical difference was found in the drug resistance detection rate among patients attending universities, secondary schools, and technical and vocational schools (χ^2^ = 2.47, *P* = 0.29). Two patients with retreated TB both had drug-resistant strains, one of which was a multidrug-resistant strain with a 100% drug resistance rate.

### 3.3. Cluster analysis

**Figure 4:**
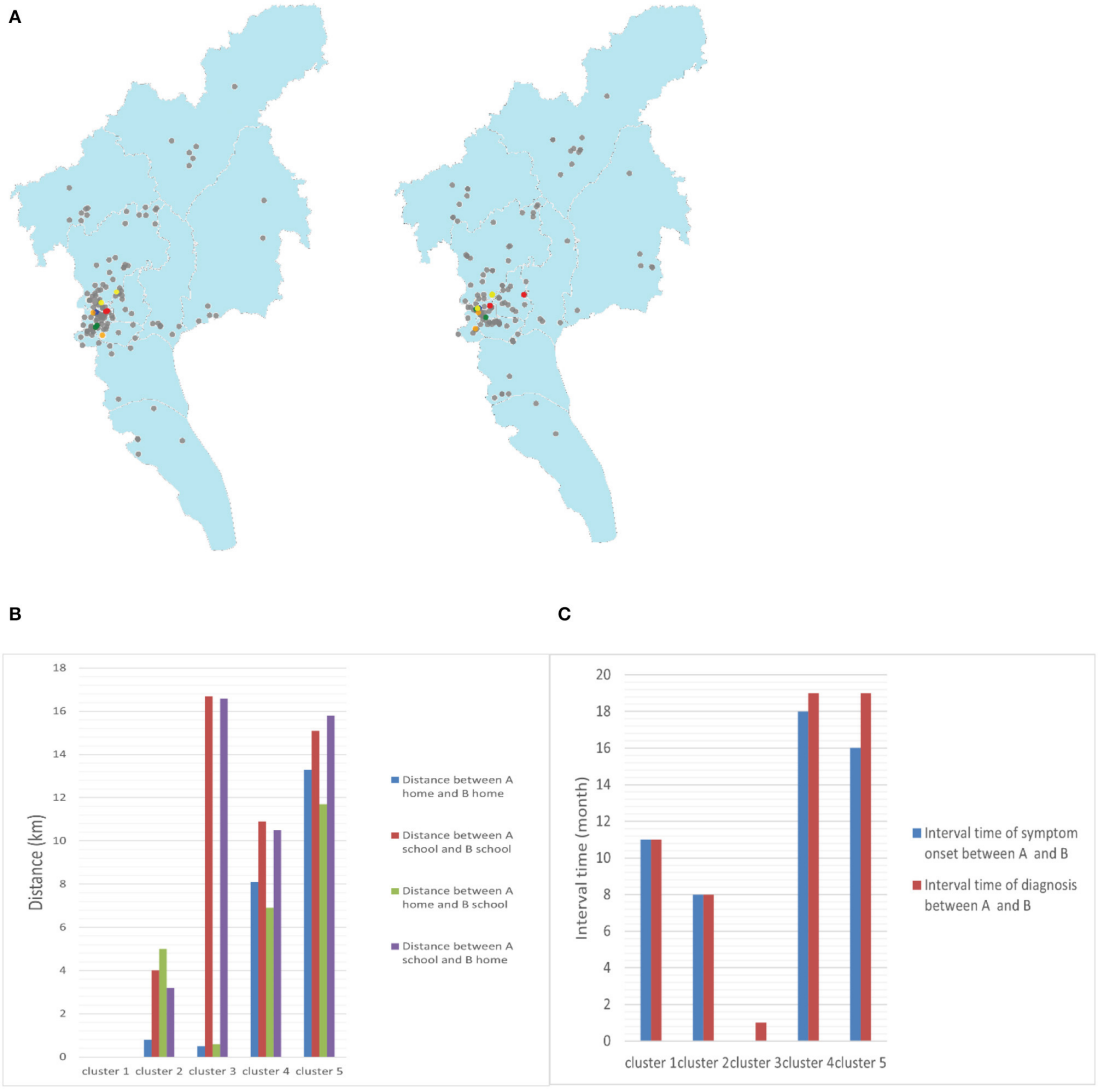
**(A)** Geographic location of the clusters in maps (blue points represent cluster 1, green points represent cluster 2, red points represent cluster 3, orange points represent cluster 4, yellow points represent cluster 5, and gray points represent non-clustered patients with TB). **(B)** Distance between the homes and schools of two patients in clusters (A and B represent two patients in one cluster, respectively). **(C)** Interval times of symptom onset and diagnosis between two patients in clusters (A and B represent two patients in one cluster, respectively).

Among the 135 isolates, we found five clusters of strains, each group containing strains from two patients, for a total of 10 cluster-forming strains with an overall cluster rate of 7.4% (10/135) (Figure 2). The home and school addresses of the 135 patients were marked on the maps. As shown in Figure 4A, the patients' homes were mainly concentrated in Baiyun district (24.44%, 33/135), Yuexiu district (19.26%, 26/135), Liwan district (13.33%, 18/135), and Haizhu district (11.85%, 16/135); of the 135 patients, 126 attended schools in Guangzhou and nine attended schools outside the city. The patients' schools were mainly distributed in Baiyun district (19.05%, 24/126), Haizhu district (13.49%, 17/126), Yuexiu district (11.90%, 15/126), and Liwan district (11.11%, 14/126). The distribution of the clustered genomes was consistent with those of the patients' home and school addresses, which were concentrated in the westward area of the Guangzhou city.

Two students in cluster 1 came from the same grade and lived in the school dormitory, and the time interval between the onset of their symptoms and diagnosis was 11 months, suggesting the possibility of intra-school transmission and a long latent epidemic with a possible intermediate chain of transmission. Cluster 2 patients lived in the same community, with a distance of only 1 km between their home addresses and interval of 8 months between symptom onset and the time of diagnosis, suggesting a high probability of community transmission and not excluding the presence of concurrent campus transmission. A similar situation existed in cluster 3. In addition, cluster 4 patients lived in different administrative districts but close to each other, approximately 8 km apart, and in the same shopping district. They may have been infected with the same or very similar local strains from community. Cluster 5 cases lived in different administrative districts, approximately 11.7 km apart, and attended different schools. The absence of direct epidemiological linkage of the patients may be due to TB infection attributable to transmission within a larger community and the presence of concurrent school transmission cannot be excluded (Figures 4B, C). From the above sequencing results, the rate of intra-school transmission with clear evidence was 1.48% (2/135).

### 3.4. Epidemiological analysis

The results of the epidemiological statistical analysis showed that 730 patients came from 474 schools, 389 schools had only 1 sputum culture positive TB patient in 2015–2019, and the remaining 85 schools had at least two sputum culture positive TB patients in 5 years. Analysis of the epidemiological association of students, using semester and school as measure units, identified 35 schools with possible intra-school transmission involving 102 students and a campus transmission rate of 14.0% (102/730).

### 3.5. Genetic distance analysis

**Figure 5:**
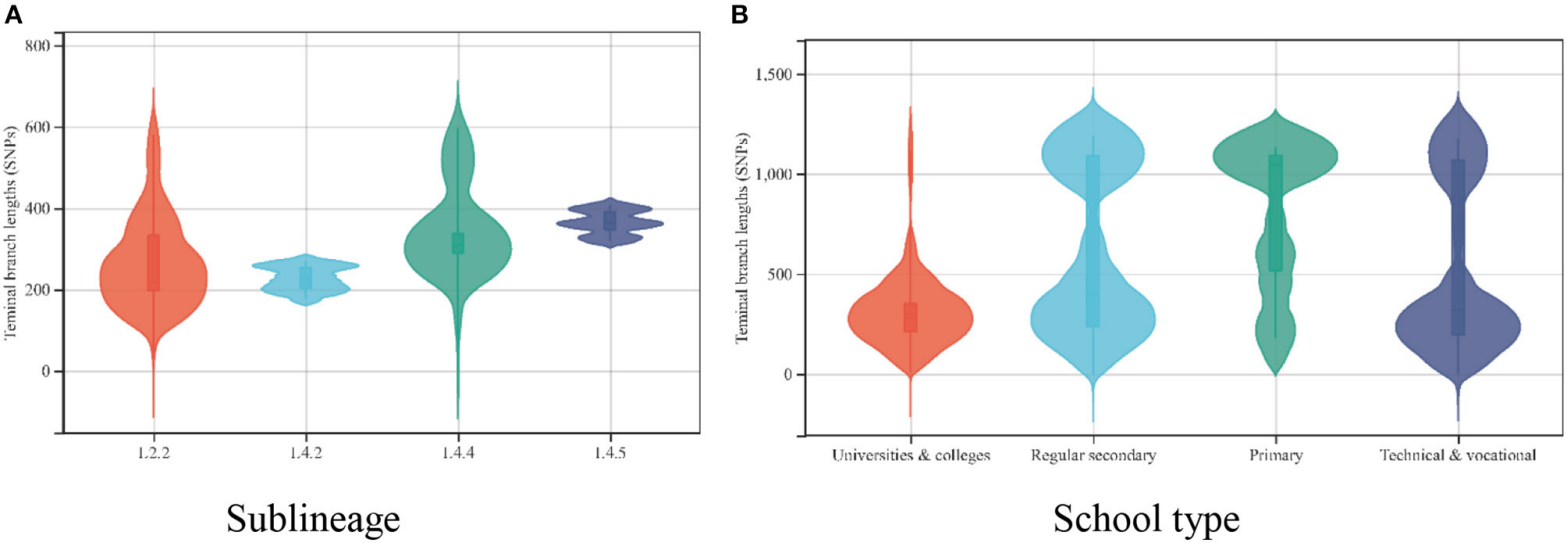
**(A)** The distribution of the terminal branch lengths for the different sublineages of 135 *Mycobacterium tuberculosis* strains. **(B)** The distribution of the terminal branch lengths for 135 *Mycobacterium tuberculosis* strains in different schools.

We calculated the TBLs of the different substrains in our study and found that the *Mycobacterium tuberculosis* strains isolated from patients attending schools in Guangzhou were dominated by long TBLs, with a median of 337 SNPs (interquartile range, 207–1075 SNPs). The median TBLs varied among strains of different sublineages, with the ancient Beijing strain (L2.2) usually having the shortest TBLs and the L4.5 strain having the longest TBLs (20). In this study, we found significantly lower TBLs within the strain L2.2 compared with the remaining strains (the median TBLs were 216 SNPs compared with 226, 310, and 362 SNPs for lineages L4.2, L4.4, and L4.5, respectively; *P* = 0.000 for all). Assuming a mutation rate of 0.3–0.5 SNPs/genome/year for *Mycobacterium tuberculosis* (15, 21), strains of different sublineages would require 674–1,123 years to accumulate 216–362 SNPs. However, this study collected patient strains from 2015 to 2019, and the strain mutation time was only 5 years, which could not be caused by transmission and mutation on campus (Figure 5A).

Moreover, we found that the TBLs varied by type of school (χ^2^ = 138.372, *P* = 0.000). *Mycobacterium tuberculosis* strains isolated from patients attending universities and colleges had the shortest TBLs, with a median of 239 SNPs (interquartile range, 201–379 SNPs), whereas primary school students had the longest TBLs, with a median of 1,038 SNPs (interquartile range, 514–1,087 SNPs) (Figure 5B).

## 4. Discussion

The incidence of TB in China is declining by approximately 4% every year, which is approximately 2 times faster than the global decline rate (13); however, TB remains an important public health concern in China. Schools have a dense population, and teachers and students are in close contact in such settings; thus, when a TB case occurs, it may spread and cause an aggregated outbreak. Thus, it is very important to elucidate the epidemic and transmission patterns of TB in schools and to apply maximum efforts to prevent and control TB in schools.

In this study, 730 sputum culture-positive students in Guangzhou city from 2015 to 2019 were performed epidemiological analysis, and a total of 135 cases from them were subjected to WGS. No statistically significant differences were found in gender, age, school type, patient source, and TB history between the samples included in WGS and those not. Statistical analysis results showed that the majority of the cases were concentrated in high schools, colleges, and technical and vocational schools, similar to that reported in a related domestic study (9). This may be related to the fact that students age 16–22 years are a susceptible group for tuberculosis. Health administrative departments and disease control departments need to focus on the prevention and control of TB among this population, and implement targeted control measures to effectively prevent and control TB. Of the included patients, the majority visited doctors voluntarily owing to an uncomfortable feeling or respiratory symptoms, and only minority of the patients were identified through health screening. It is necessary to strengthen physical examinations and implement close contact screening to improve the timely detection of TB among students.

The WGS results of the strains revealed drug-resistant mutations and helped to estimate the rate of drug resistance to anti-tuberculosis drugs in the population. The study results showed that the genotypic drug resistance rate of the *Mycobacterium tuberculosis* culture-positive patients attending schools in Guangzhou was 16.3%, with the college student population accounting for the largest proportion (25.81%), and that the rate of multidrug resistance was 6.6%, which was similar to that reported of Shenzhen (19.6%) (13) and Luodian (14.0%) (22) in southern China. The incidence of drug resistance in two patients with retreated TB was 100%, which was significantly higher than the rate of drug resistance in the new patients. Owing to the dense population and high rates of drug resistance in South China, drug resistance screening among *Mycobacterium tuberculosis* culture-positive patients needs to be strengthened to detect drug-resistant patients promptly and to perform targeted individualized treatment. Treatment management of infected students should simultaneously be standardized to improve the effectiveness of governance and reduce the number of relapse cases.

WGS can precisely distinguish clusters of genetically related isolates and identify the associations among cases with limited genomic information on the strain (5, 6, 23). However, due to a small sample size of whole genome sequencing in our study, complete chains of transmission were hard to construct. Consequently, we used WGS combined with epidemiological analysis of TB to explore the transmission patterns in patients attending schools in Guangzhou. Cluster analysis revealed that the genome cluster rate of the 135 patients with TB was 7.4%, which is much lower than the proportion of TB transmission in China (31%) (24) and some nearby Cities of Shenzhen (12.1%) (13), Luodian (13.1%) (22), Yichang (31.1%) (25), and Shanghai (31.8%) (15). Additionally, epidemiological analysis identified 35 schools with possible intra-school transmission involving 102 students in 730 patients with positive sputum culture, which got the intra-school transmission rate to be 14.0%. The epidemiological results can explain the transmission relationship partly. However, cases from the same school with closed diagnosis times (<6 months) may not due to intra-school transmission, they may come from community infection. The rate (14%) may overestimates the actual intra-school transmission value. Combining the results of whole genome sequencing and traditional epidemiology, we inferred that the campus transmission rate of school TB in Guangzhou city was between 1.48 and 14.0%. Although the rates may not be fully comparable owing to differences in the samples included and the methods used to assess the transmission patterns, the lower rate noted in this study still suggests that intra-school transmission has a relatively small contribution to the overall TB burden in educational settings in Guangzhou. Among the clustered genomes, only 1.48–14% had a clear history of epidemiological transmission from school, suggesting that the occurrence of TB among patients attending schools in Guangzhou is mainly attributable to community transmission. The focus of TB prevention and control should be on disease surveillance, including medical examination for new students and daily surveillance. Moreover, adequate prevention and control of TB in the whole population and an effective reduction in the TB incidence rate in the population are necessary to effectively control the transmission of TB among those attending schools.

In addition, in this study, the average interval time of symptom onset and diagnosis between patients in clusters was 11 months, which significantly exceeded the incubation period of TB by 2–3 months, suggesting that the infected strains were transmitted in the population over long periods. In a study of TB events in family outbreaks, British scholars (22) found that the rate of change in the DNA sequence was approximately 0.5 SNPs/genome/year and that patients with epidemiological associations were genetically linked by ≤ 5 SNPs. In this study, the distance between the strains among cases clustered in schools was eight SNPs, with an interval of 11 months between symptom onset in patient 1 (October 2016) and patient 2 (September 2017), indicating that the strain was transmitted in the school over several generations. The results of analysis of the spatial and temporal characteristics of TB incidence in the relevant local school (10) revealed that a campus cluster outbreak occurred in the school between September 2017 and April 2018, with 12 students contracting TB. Patient 2 was one of the cases in this cluster outbreak, and strains from the remaining 11 students could not be obtained due to the retention and recovery of the strains. Access to the on-site epidemiological investigation of the outbreak at the Guangzhou Chest Hospital revealed a clear epidemiological association among the 12 students. In summary, TB transmission occurred in at least 13 students at the school between October 2016 and April 2018. The spread of TB, which was not controlled promptly, resulted in an aggregated outbreak after 11 months.

Based on the above findings, TB outbreaks in Guangzhou schools are mainly disseminated, but occasional aggregated outbreaks may occur. In a case of negligent control, an epidemic can easily turn into an outbreak; therefore, it is crucial to strengthen TB surveillance and implement close contact screening in schools. Moreover, schools should conduct timely morning checks, tracking of absenteeism due to illness, and etiology tracing to strengthen the surveillance of TB among students to achieve early TB detection. Disease prevention and control institutions should strengthen disease surveillance and conduct timely screening of close contacts to stop the spread of TB epidemics in schools.

This study has certain limitations. First, the number of TB cases caused by transmission in schools may have been underestimated. Because only patients with positive sputum cultures were considered for inclusion in this study, those with negative sputum cultures or unsuccessful re-culturing of frozen stocks and limited information on their strains could not be assessed, resulting in the absence of certain *Mycobacterium tuberculosis* transmission links and failure to form a complete transmission chain. Second, because we only analyzed strains isolated from patients with TB attending schools and diagnosed at the Guangzhou Chest Hospital, we were unable to gain insights into the transmission patterns of TB in the entire city owing to biases in the selection of the attendees and study participants; thus, our findings from patients with TB attending schools may not be generalizable to other populations.

Overall, the whole-genome sequences and epidemiological analysis result clarified that the transmission of TB among schools in Guangzhou was mainly disseminated, which was attributable to community imported, and transmission with aggregated outbreaks occur occasionally. We also clarified the future directions and measures for TB prevention and control. We suggest the use of genomic data in combination with basic clinical and epidemiological data to identify aggregated TB cases and infer some potential sources when investigating TB outbreaks in schools and other closed and semi-closed settings. Combining WGS results with extensive data to obtain a more comprehensive view of TB transmission can help conduct detailed epidemiological analyses of TB transmission.

## Data availability statement

The original contributions presented in the study are included in the article/Supplementary material, further inquiries can be directed to the corresponding author/s.

## Ethics statement

The studies involving human participants were reviewed and approved by Ethics Review Committee of the Guangzhou Chest Hospital (No. 2019045). Written informed consent to participate in this study was provided by the participants' legal guardian/next of kin.

## Author contributions

YLin and YD: project administration, methodology, formal analysis, and writing-original draft. HS: formal analysis and writing-original draft. YG, TW, KL, DZ, GZ, GW, and YLei: formal analysis. JL: conceptualization, writing-review and editing, and supervision. All authors contributed to the article and approved the submitted version.
