# Supplementary material for: Transmission of Mycobacterium tuberculosis in schools: a molecular epidemiological study using whole-genome sequencing in Guangzhou, China
Source: Front Public Health. 2023 May 11;11:1156930. doi: 10.3389/fpubh.2023.1156930 (PMC10219607; doi:10.3389/fpubh.2023.1156930)
Supplement: Supplementary file 2 [file Table_2.docx]

Supplementary Table 2 Analysis of whole gene sequencing results

|  | No. | % |
| --- | --- | --- |
| **Genetype** |  |  |
| L2.1 | 1 | 0.7% |
| L2.2 | 106 | 78.5% |
| L4.2 | 5 | 3.7% |
| L4.4 | 17 | 12.6% |
| L4.5 | 6 | 4.4% |
| **Drug resistance type** |  |  |
| Mono-drug resistance | 8 | 5.9% |
| Multi-drug resistance | 8 | 5.9% |
| Extensively-drug resistance | 1 | 0.7% |
| Other drug resistance | 5 | 3.7% |
| Pan Sensitive | 113 | 83.7% |
| **Drug resistance of drugs** |  |  |
| isoniazid | 15 | 11.11% |
| streptomycin | 11 | 8.15% |
| rifampicin | 10 | 7.41% |
| moxifloxacin | 9 | 6.67% |
| ofloxacin | 9 | 6.67% |
| pyrazinamide | 5 | 3.7% |
| ethambutol | 3 | 2.2% |
| ethionamide | 1 | 0.7% |
| amikacin | 1 | 0.7% |
| capreomycin | 1 | 0.7% |
| kanamycin | 1 | 0.7% |
| para-aminosalicylic acid | 0 | - |
| cycloserine | 0 | - |
| linezolid | 0 | - |
| clofazimine | 0 | - |
| bedaquiline | 0 | - |
| delamanid | 0 | - |
